# Supplementary material for: The association between physical activity and erectile dysfunction: A cross-sectional study in 20,789 Brazilian men
Source: PLoS One. 2022 Nov 16;17(11):e0276963. doi: 10.1371/journal.pone.0276963 (PMC9668147; doi:10.1371/journal.pone.0276963)
Supplement: S1 Appendix — (DOCX) [file pone.0276963.s003.docx]

| S2 Appendix. Predictors of ED (n=20,789). | | | | |  |
| --- | --- | --- | --- | --- | --- |
| **Variables** | **OR** | **CI (95%)** | | **p** | |
| Age | 1,09 | 1,08 | 1,09 | <0,001 |  |
| Hypertension | 1,19 | 1,08 | 1,31 | 0,001 |  |
| Diabetes mellitus | 1,37 | 1,20 | 1,58 | <0,001 |  |
| Dyslipidemia | 1,06 | 0,96 | 1,16 | 0,245 |  |
| Body mass index | 1,03 | 1,02 | 1,04 | <0,001 |  |
| Metabolic Syndrome | 1,01 | 0,88 | 1,17 | 0,876 |  |
| Tobacco use |  |  |  |  |  |
| *Previous* | 0,90 | 0,81 | 0,99 | 0,034 |  |
| *Active* | 1,14 | 0,99 | 1,32 | 0,069 |  |
| Physical Activity Level |  |  |  |  |  |
| *Low active* | 0,77 | 0,68 | 0,87 | <0,001 |  |
| *Moderate* | 0,91 | 0,81 | 1,01 | 0,086 |  |
| *High Active* | 0,84 | 0,71 | 0,98 | 0,03 |  |
| Non-alcoholic fatty liver diease | 0,94 | 0,86 | 1,04 | 0,237 |  |
| Lower urinary tract symptoms |  |  |  | 0 |  |
| *Moderate* | 2,75 | 2,43 | 3,12 | <0,001 |  |
| *Severe* | 3,10 | 2,23 | 4,30 | <0,001 |  |
| Alcohol consumption |  |  |  |  |  |
| *Intermediary* | 1,06 | 0,94 | 1,19 | 0,323 |  |
| *High* | 1,11 | 0,85 | 1,45 | 0,455 |  |
| Perceived Stress | 1,09 | 0,97 | 1,22 | 0,13 |  |
| Depressive symptoms | 2,10 | 1,86 | 2,38 | <0,001 |  |
| HDL | 0,99 | 0,99 | 1,00 | 0,001 |  |
| Triglycerides | 1,00 | 1,00 | 1,00 | 0,17 |  |
| LDL | 1,00 | 1,00 | 1,00 | 0,001 |  |
| Full multiple logistic regression | | | | |  |
